# The Impact of Idiopathic Intracranial Hypertension on Cardiovascular Disease Risk Among UK Women: An Obesity-Adjusted Analysis

**DOI:** 10.71079/h1fr8h68

**Published:** 2024-11-17

**Authors:** Ahmed Y. Azzam, Mahmoud M. Morsy, Mohamed Hatem Ellabban, Ahmed M. Morsy, Adham Adel Zahran, Mahmoud Nassar, Omar S. Elsayed, Adam Elswedy, Osman Elamin, Ahmed Saad Al Zomia, Hana J. Abukhadijah, Hammam A. Alotaibi, Oday Atallah, Mohammed A. Azab, Muhammed Amir Essibayi, Adam A. Dmytriw, Mohamed D. Morsy, David J. Altschul

**Affiliations:** 1- Faculty of Medicine, October 6 University, 6^th^ of October City, Giza, Egypt.; 2- Montefiore-Einstein Cerebrovascular Research Lab, Albert Einstein College of Medicine, Bronx, NY, USA.; 3- Director of Clinical Research and Clinical Artificial Intelligence, American Society for Inclusion, Diversity, and Health Equity (ASIDE), Delaware, USA.; 4- Clinical Research Fellow, American Society for Inclusion, Diversity, and Health Equity (ASIDE), Delaware, USA.; 5- Faculty of Medicine, Al-Azhar University, Cairo, Egypt.; 6- Kasr Alainy Faculty of Medicine, Cairo University Hospitals, Cairo University, Cairo, Egypt.; 7- Department of Medicine, Division of Endocrinology, Diabetes and Metabolism, Jacobs School of Medicine and Biomedical Sciences, University at Buffalo, New York, USA.; 8- Founder, American Society for Inclusion, Diversity, and Health Equity (ASIDE), Delaware, USA.; 9- Department of Neurosurgery, Jordan Hospital, Amman, Jordan.; 10- College of Medicine, King Khalid University, Abha, Saudi Arabia; 11- Medical Research Center, Hamad Medical Corporation, Doha, Qatar.; 12- Ophthalmology Department, Prince Sultan Military Medical City, Riyadh, Saudi Arabia.; 13- Department of Neurosurgery, Hannover Medical School, Hannover, Germany.; 14- Department of Neurosurgery, Cleveland Clinic Foundation, Cleveland, OH, USA.; 15- Department of Neurological Surgery, Montefiore Medical Center, Albert Einstein College of Medicine, Bronx, NY, USA.; 16- Neuroendovascular Program, Massachusetts General Hospital & Brigham and Women’s Hospital, Harvard University, Boston, MA, USA; 17- Neurovascular Centre, Divisions of Therapeutic Neuroradiology & Neurosurgery, St. Michael’s Hospital, University of Toronto, Toronto, ON, Canada.; 18- College of Medicine, King Khalid University, Abha, Saudi Arabia.

**Keywords:** Idiopathic Intracranial Hypertension, Pseudotumor Cerebri, Stroke, Ischemic Stroke, Cardiovascular Disease

## Abstract

**Introduction:**

Idiopathic intracranial hypertension (IIH) is known to elevate cardiovascular disease (CVD) risk, but the extent to which obesity and IIH-specific factors contribute to this risk is not well understood. WE aim to separate the effects of obesity from IIH-specific factors on the risk of stroke and CVD, building on previous findings that indicate a two-fold increase in cardiovascular events in women with IIH compared to BMI-matched controls.

**Methods:**

An obesity-adjusted risk analysis was conducted using Indirect Standardization based on data from a cohort study by Adderley et al., which included 2,760 women with IIH and 27,125 matched healthy controls from The Health Improvement Network (THIN). Advanced statistical models were employed to adjust for confounding effects of obesity and determine the risk contributions of IIH to ischemic stroke and CVD, independent of obesity. Four distinct models explored the interactions between IIH, obesity, and CVD risk.

**Results:**

The analysis showed that IIH independently contributes to increased cardiovascular risk beyond obesity alone. Risk ratios for cardiovascular outcomes were significantly higher in IIH patients compared to controls within similar obesity categories. Notably, a synergistic effect was observed in obese IIH patients, with a composite CVD risk ratio of 6.19 (95% CI: 4.58–8.36, p<0.001) compared to non-obese controls.

**Conclusions:**

This study underscores a significant, independent cardiovascular risk from IIH beyond obesity. The findings advocate for a shift in managing IIH to include comprehensive cardiovascular risk assessment and mitigation. Further research is required to understand the mechanisms and develop specific interventions for this group.

## 1. Introduction

Idiopathic intracranial hypertension (IIH) is a condition characterized by elevated intracranial pressure of unknown etiology, typically manifesting as papilledema with associated risks of visual loss and chronic disabling headache [1]. The incidence and economic burden of IIH are rising in parallel with global obesity trends [2]. While obesity is a well-established risk factor for IIH, with over 90% of patients being obese [3], the relationship between IIH and cardiovascular disease (CVD) risk remains poorly understood.

In the United States, studies indicate an incidence increase from 1.6 to 2.4 per 100,000 person-years in the general population, rising dramatically to 15–19 per 100,000 in women of childbearing age [4]. This rising disease burden encompasses both economic impacts, with annual costs exceeding millions of dollars in the US [5], and significant quality of life deterioration, including chronic pain, vision problems, and psychological distress [6].

Adderley et al. conducted a retrospective case-control population-based matched controlled cohort study using 28 years of data from The Health Improvement Network (THIN) database in the United Kingdom, THIN database is a longitudinal primary care database containing anonymized electronic health records from over 17 million patients in the United Kingdom, provides researchers with comprehensive clinical data for epidemiological studies and healthcare research. [7]. Their study suggested that women with IIH have a two-fold increased risk of cardiovascular events compared to BMI-matched controls. However, the mechanisms underlying this elevated risk and the relative contributions of obesity versus IIH-specific factors remained unclear.

The relationship between IIH and CVD risk involves multiple pathophysiological mechanisms beyond adiposity alone. Neuroendocrine dysfunction in IIH is characterized by elevated endogenous testosterone and androstenedione levels [8], distinct from exogenous supplementation or polycystic ovary syndrome (PCOS). This hormonal dysregulation may affect both cerebrospinal fluid (CSF) dynamics and cardiovascular function [9]. Additionally, the current literature studies demonstrate elevated levels of pro-inflammatory cytokines in IIH patients, potentially contributing to both intracranial pressure elevation and vascular dysfunction [9]. IIH patients exhibit distinct metabolic profiles, including altered glucose homeostasis and lipid metabolism, which may independently contribute to cardiovascular risk [9, 10]. Several additional risk factors may contribute to both IIH and CVD, including hormonal contraceptive use, vitamin A metabolism, sleep apnea, and chronic kidney disease [10–12].

Building upon Adderley et al.’s [7] findings, our study aims to disentangle the effects of obesity and IIH on stroke risk specifically. Obesity is a known independent risk factor for stroke, with an average hazard ratio (HR) of 2.29 reported in large-scale evidence [13]. By adjusting for this obesity-related risk, we seek to isolate the potential contribution of IIH itself to stroke incidence.

Our study employs an established methodological approach adapted from epidemiological research in obesity [14, 15] to simulate predicted ischemic stroke and CVD events in both IIH and control groups under normative weight conditions. This approach has been previously used in obesity literature [16, 17].

Understanding the relationship between IIH and their associated risks, independent of obesity, has important clinical implications. If IIH itself confers additional cardiovascular risk, it may warrant more aggressive management of modifiable risk factors and earlier implementation of preventive strategies in this patient population. Furthermore, elucidating the mechanisms underlying this potential association could reveal new therapeutic targets for reducing cardiovascular morbidity in IIH. Our study aims to build upon the foundational work of Adderley et al. [7] to further investigate the complex interplay between IIH, obesity, and the associated risks. By employing innovative statistical methods to adjust for the confounding effects of obesity, we aim to provide crucial insights into the cardiovascular implications of IIH and inform evidence-based management strategies for this increasingly prevalent condition.

## 2. Methods

Building upon the foundational work of Adderley et al. [7], we conducted a retrospective analysis using data from their paper which was originally obtained through THIN, a large UK primary care database. Our study focused on women with IIH and matched controls, aiming to elucidate the independent effect of IIH on stroke and cardiovascular risks, distinct from the influence of obesity. Patients were excluded from the Adderley et al. [7], study if they had different diagnostic or clinical codes for conditions that could mimic IIH, specifically hydrocephalus or cerebral venous thrombosis, or any other cause of elevated intracranial pressure (ICP).

Additionally, in the baseline cohort selection, female patients were excluded if they did not have at least one-year of registration with an eligible general practice before cohort entry, to ensure adequate documentation of baseline covariates. For the analysis of individual CVD outcomes, patients with a record of the specific outcome of interest at baseline were excluded from the corresponding analysis, for composite CVD analysis, patients with any CVD at baseline were excluded; for type 2 diabetes analysis, patients with either type 1 diabetes or type 2 diabetes at baseline were excluded. For sensitivity analyses, additional exclusions were applied, including excluding women diagnosed with IIH after age 60 years, since IIH is rare among older adults and there may be potential misclassification errors in this age group.

### 2.1. Study Population and Data Source:

We utilized the cohort established by Adderley et al. [7], comprising 2,760 women with IIH and 27,125 matched controls. Participants were identified from THIN database records spanning January 1, 1990, to January 17, 2018. Controls were matched to IIH patients based on age, body mass index (BMI), and sex, with up to 10 controls per IIH case.

### 2.2. Outcome Measures:

Our primary outcome of interest was the incidence of composite CVD, heart failure, ischemic heart disease (IHD), ischemic stroke, transient ischemic attack (TIA), hypertension, and type 2 diabetes mellitus. We extracted the relevant data from the corresponding paper, following the coding and identification methods described by Adderley et al [7].

### 2.3. Statistical Analysis:

We extended the original analysis to estimate the independent effect of IIH on stroke and cardiovascular risks, accounting for the confounding effect of obesity. Our approach involved indirect standardization and adjustment with the application of a standardized morbidity ratio (SMR) approach [18–22], adapted to account for obesity as a confounding variable in relationship with IIH in women around the UK. To estimate the incidence of events in both the IIH and control cohorts under a hypothetical scenario of normal weight, we employed an adjustment method based on the average HR for obesity contributing to the event risk in women compared to healthy weight women in 13-year interval from the literature. This approach operates under the assumption that the HR remains constant throughout the 13-year study period and that the impact of obesity on the estimated events is independent of IIH status. We utilized Python 3.12 and its’ associated statistical libraries to perform our statistical analysis. Initially, we calculated the observed HR for each event in the IIH group compared to the control group. Subsequently, we adjusted this observed HR by obesity HR to estimate the HR for IIH independent of obesity. Based on the current evidence, the average estimated HR of obesity contributing to composite CVD is 2.89 [23–29]. For obesity, ischemic stroke, and TIA risk, it is estimated around HR= 1.72 [23, 26, 30–36]. For obesity and heart failure risk, it is estimated around HR= 2.61 [37–43]. For obesity and hypertension risk, it is estimated around HR= 2.09 [44–50]. For obesity and IHD risk, it is estimated around HR= 1.8 [23, 24, 26, 28, 30, 51, 52]. And for obesity and type 2 diabetes mellitus risk, it is estimated to be around HR= 4.0 [53–60].

We calculated the HR for each event in the IIH group compared to the control group through the following equation:

HR=(IIHevents/IIHtotal)/(Controlevents/Controltotal)

We then adjusted this observed HR by the established HR for obesity in association with the potential risk to estimate the HR for IIH independent of obesity:

AdjustedHR=ObservedHR/ObesityHR

Using this adjusted HR, we predicted the number of events in both groups under normative weight conditions:

For the IIH group:

PredictedIIHevents=(AdjustedHR×Controlevents×IIHtotal)/Controltotal

For the control group:

PredictedControlevents=Controlevents/ObesityHR

Using this adjusted HR, we then calculated the predicted number of events in both the IIH and control groups under the assumption of normal weight. This was accomplished by applying the adjusted HR to the control group event rate and scaling for the respective group sizes. For the control group, we divided the observed events by obesity HR to estimate events under normal weight conditions.

This method allows for a comparative analysis of events risk between IIH and control populations, while attempting to control the confounding effect of obesity. It provides insight into the potential independent risk associated with IIH and allows for estimation of event rates under hypothetical normal weight conditions.

### 2.4. Ethical Considerations:

This study adhered to the ethical approval obtained by Adderley et al. [7] from the NHS South-East Multicenter Research Ethics Committee. We did not involve direct analysis of the dataset rather than building customized statistical modelling based on the provided data and metrics from Adderley et al. research paper [7].

## 3. Results

### 3.1. Baseline Characteristics:

The original retrospective cohort study by Adderley et al. [7] encompassed 29,885 participants, stratified into 2,760 (9.2%) women with IIH and 27,125 (90.8%) controls. The incident cohort comprised 48.2% and 46.7% of the IIH and control groups, respectively. Both cohorts were predominantly under 60 years of age (98.1% IIH, 95.2% control), with identical median ages of 32.1 years (IQR: 25.62–42.00 IIH, 25.71–42.06 control). Socioeconomic status, assessed via Townsend Deprivation Quintiles, showed a comparable distribution between groups, with a slight overrepresentation of controls in the least deprived quintiles. Smoking habits differed significantly: the IIH cohort exhibited higher rates of current smoking (30.8% vs 22.6%) and lower rates of non-smoking (46.5% vs 55.5%).

Anthropometric data revealed marginally higher median BMI in the IIH group (34.80, IQR: 29.30–40.30) compared to controls (34.30, IQR: 29.00–39.70). Notably, both groups demonstrated a high prevalence of obesity (BMI >30), affecting 62.6% and 60.9% of the IIH and control cohorts, respectively. Comorbidity profiles and pharmacological interventions showed distinct patterns. The IIH cohort demonstrated a higher prevalence of migraine (21.0% vs. 11.9%), hypertension (13.8% vs. 9.2%), and marginally increased rates of lipid-lowering medication use (6.5% vs. 5.8%). Furthermore, baseline cardiovascular morbidity was more pronounced in the IIH group, with elevated rates of ischemic heart disease (1.3% vs. 0.9%) and ischemic stroke/TIA (1.7% vs 0.7%). Interestingly, type 2 diabetes mellitus prevalence was slightly lower in the IIH cohort (4.7% vs. 5.2%) (Table 1).

### 3.2. Statistical Analysis:

In this analysis, we employed four distinct statistical models to elucidate the complex interrelationships between IIH, obesity, and CVD risk. These models were strategically designed to disentangle the individual and combined effects of IIH and obesity on CVD outcomes.

Model 1 (Obese IIH vs Obese Control) was constructed to isolate the effect of IIH within an obese population, effectively controlling for the confounding factor of adiposity. Model 2 (Obese IIH vs Non-obese Control) provided a comprehensive view of the combined impact of IIH and obesity compared to individuals without either condition. Model 3 (Non-obese IIH vs Obese Control) offered a unique perspective, juxtaposing the cardiovascular risks associated with IIH in non-obese individuals against those attributed to obesity alone. Model 4 (Non-obese IIH vs. Non-obese Control) isolated the impact of IIH in a non-obese population, providing critical insights into the condition’s effects independent of obesity (Table 2).

**Figure 1:**
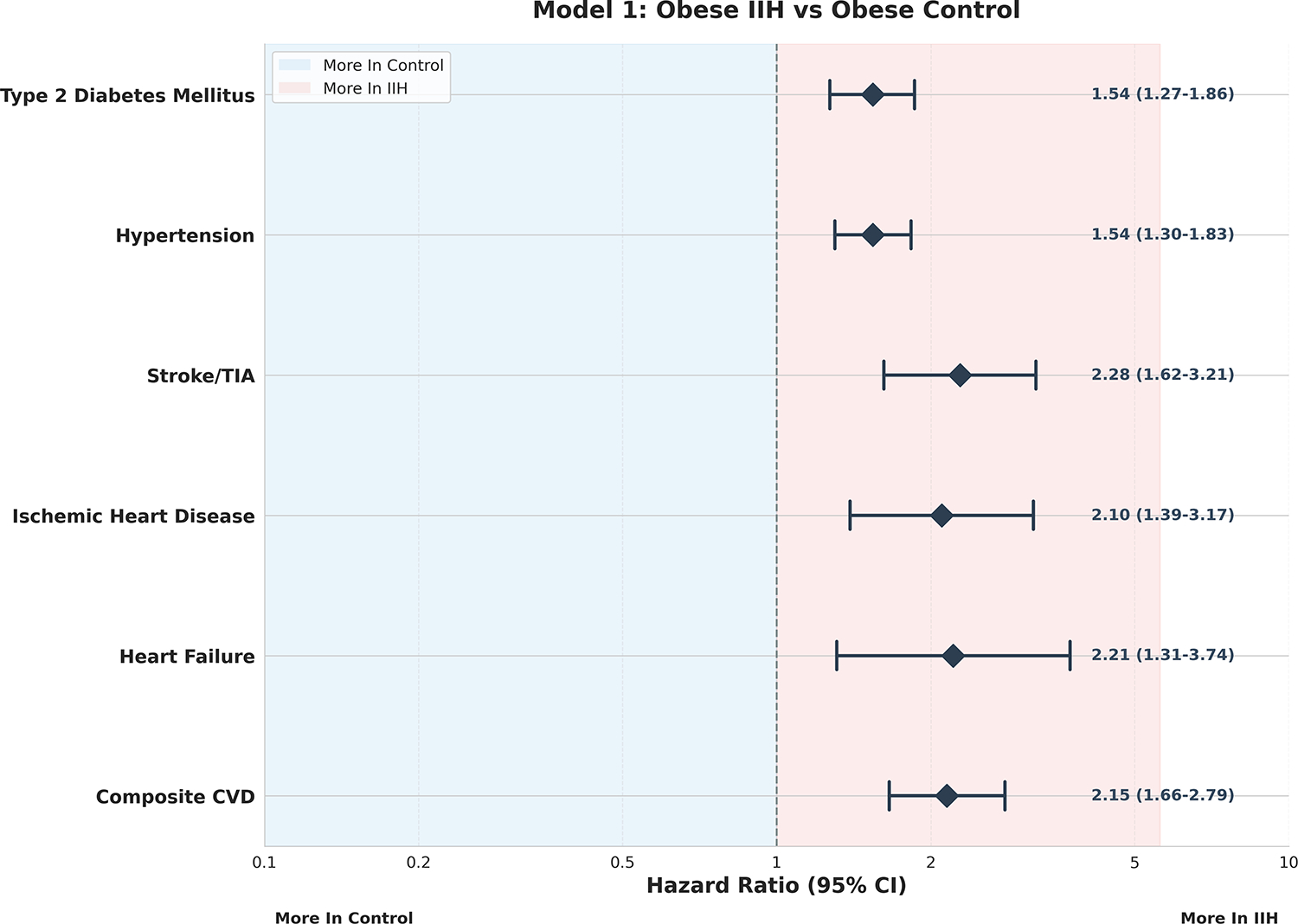
Model 1 – Obese IIH vs Obese Control Forest Plot.

Our findings revealed a nuanced and clinically significant relationship between IIH, obesity, and cardiovascular risk. In Model 1 Figure 1, IIH was consistently associated with elevated risks across all measured outcomes. The risk ratios (RR) ranged from 1.54 (95% CI: 1.27–1.86, p<0.001) for type 2 diabetes mellitus to 2.28 (95% CI: 1.62–3.21, p<0.001) for stroke/TIA. This uniform pattern of risk elevation suggests that IIH confers additional cardiovascular risk beyond that attributed to obesity alone, a finding of relevance in clinical risk stratification.

**Figure 2:**
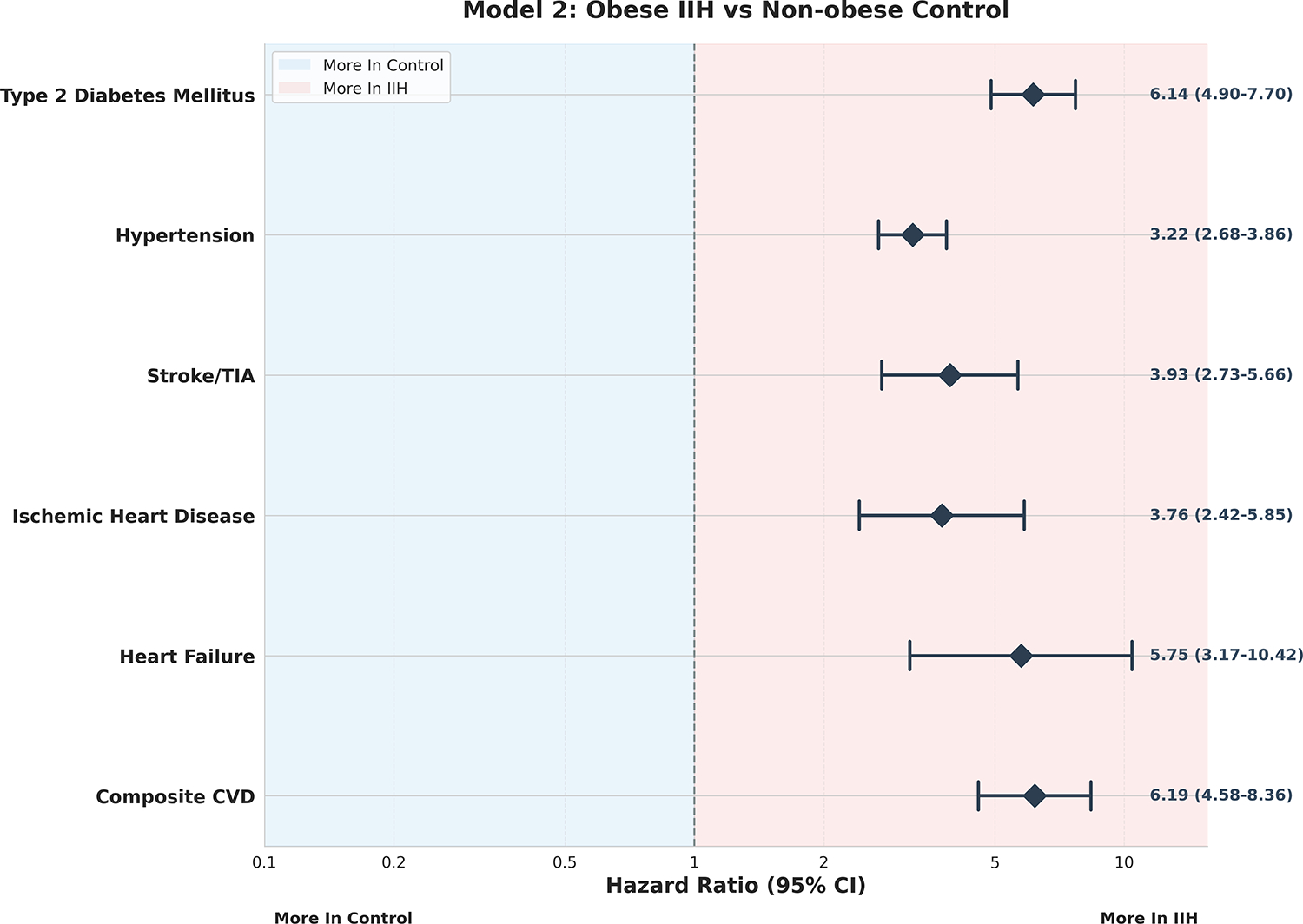
Model 2 – Obese IIH vs Non-Obese Control Forest Plot.

Model 2, Figure 2 demonstrated even more pronounced risk elevations, with the composite CVD risk reaching a striking RR of 6.19 (95% CI: 4.58–8.36, p<0.001). This marked increase suggests a potential synergistic effect between IIH and obesity on cardiovascular health, which may have significant implications for patient management and therapeutic interventions. Notably, the risk for heart failure in this model was particularly elevated (RR 5.75, 95% CI: 3.17–10.42, p<0.001), highlighting the need for vigilant cardiac monitoring in obese IIH patients.

**Figure 3:**
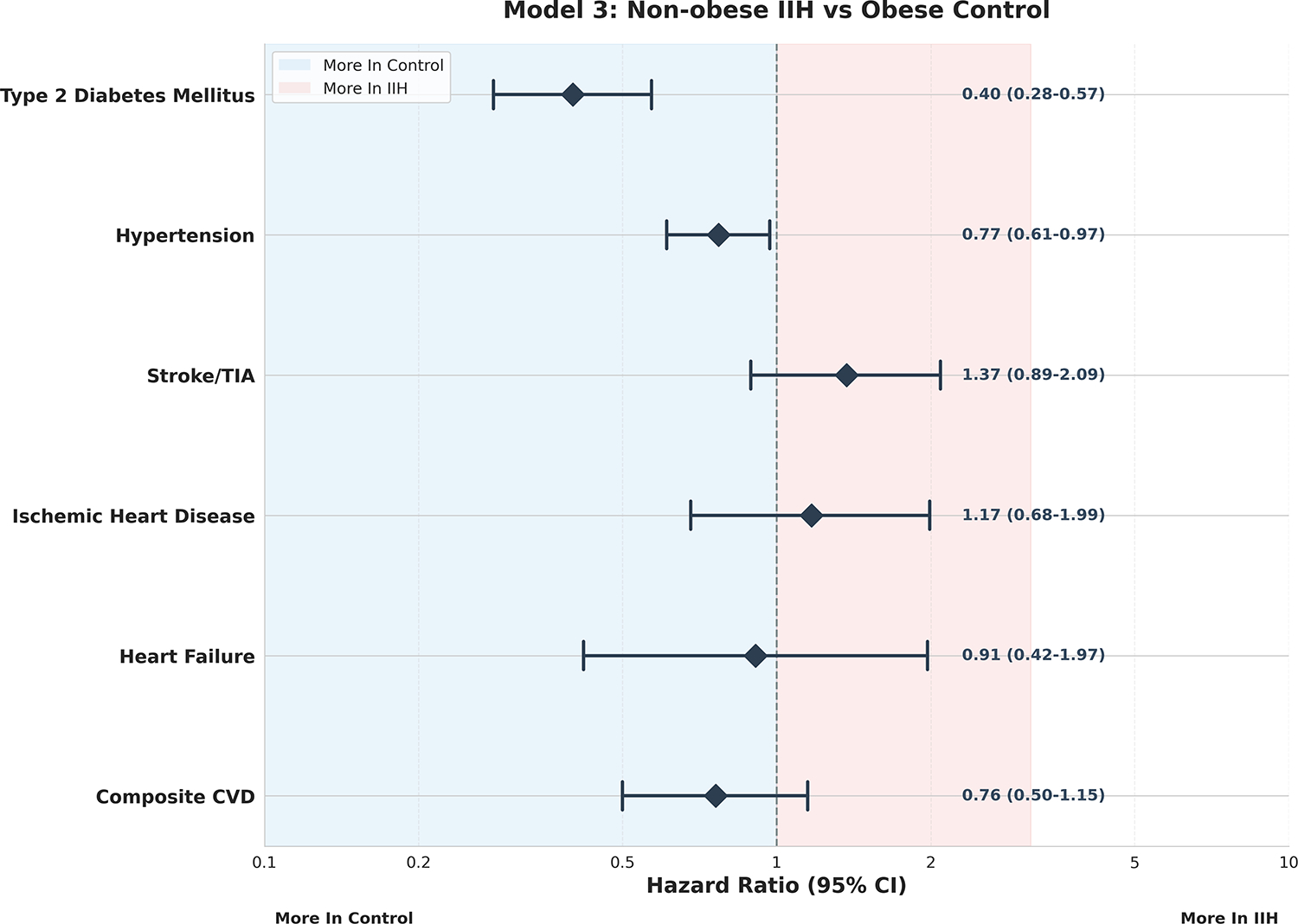
Model 3 – Non-Obese IIH vs Obese Control Forest Plot.

Interestingly, Model 3, Figure 3, presented a more complex picture. The non-significant risk ratios for most outcomes in this model suggest that non-obese individuals with IIH may not have significantly different CVD risks compared to obese individuals without IIH. This finding underscores the profound impact of obesity on cardiovascular health, potentially rivaling or even overshadowing the effects of IIH in certain contexts. Of note in this model was the significantly reduced risk of type 2 diabetes mellitus in non-obese IIH patients compared to obese controls (RR 0.40, 95% CI: 0.28–0.57, p<0.001). This intriguing paradox may offer valuable insights into the underlying pathophysiology of both conditions and warrants further mechanistic investigation.

**Figure 4:**
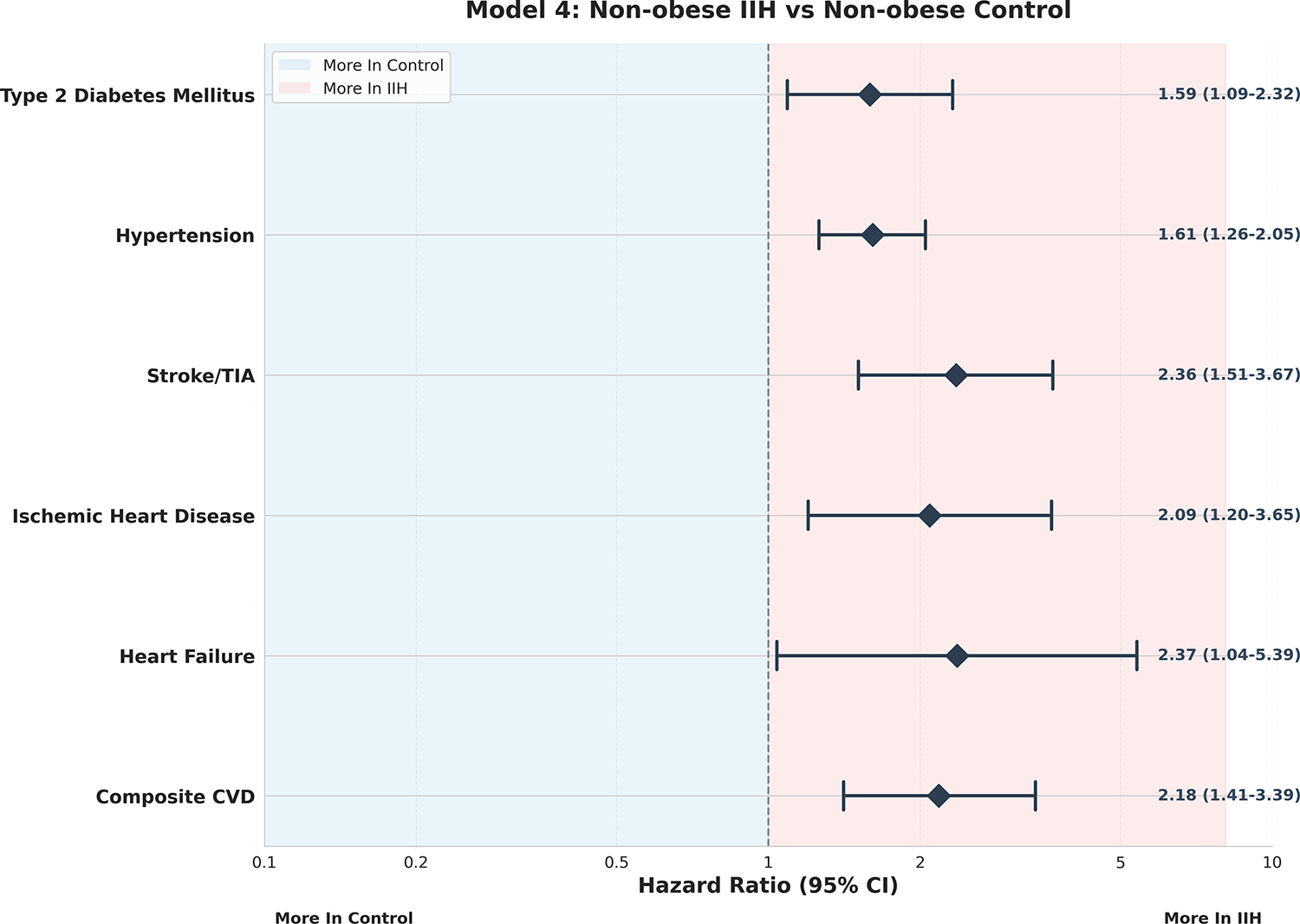
Model 4 – Non-Obese IIH vs Non-Obese Control Forest Plot.

Model 4, Figure 4 provided robust corroboration of IIH as an independent risk factor, with significant risk elevations observed across all outcomes in non-obese IIH patients compared to non-obese controls. The composite CVD risk in this model (RR 2.18, 95% CI: 1.41–3.39, p<0.001) closely mirrored that observed in Model 1, further supporting the notion that IIH confers cardiovascular risk independent of obesity status. This finding has important implications for the management of non-obese IIH patients, who may be at underappreciated cardiovascular risk.

Ranking the CVD risks for IIH patients based on our data reveals the highest risk ratios in Model 2, with the following hierarchy: composite CVD (RR 6.19) > heart failure (RR 5.75) > stroke/TIA (RR 3.93) > ischemic heart disease (RR 3.76). This stratification underscores the critical importance of addressing both IIH and obesity in our highest-risk patients and may inform the development of targeted screening and intervention protocols. The data on type 2 diabetes mellitus warrant special consideration. The 6.14-fold increased risk (95% CI: 4.90–7.70, p<0.001) observed in obese IIH patients compared to non-obese controls (Model 2) is particularly striking. This marked elevation, coupled with the paradoxical risk reduction in non-obese IIH patients (Model 3), suggests a complex interplay between IIH, obesity, and metabolic dysfunction. These findings raise intriguing questions about potential shared pathophysiological mechanisms and may open new avenues for research into the neuroendocrine aspects of IIH. Hypertension, a known risk factor for both CVD and IIH progression, showed a consistent pattern of elevated risk across Models 1, 2, and 4. However, the reduced risk observed in Model 3 (RR 0.77, 95% CI: 0.61–0.97, p=0.03) adds another layer of complexity to our understanding of the relationship between IIH, obesity, and blood pressure regulation.

## 4. Discussion

**Figure 5:**
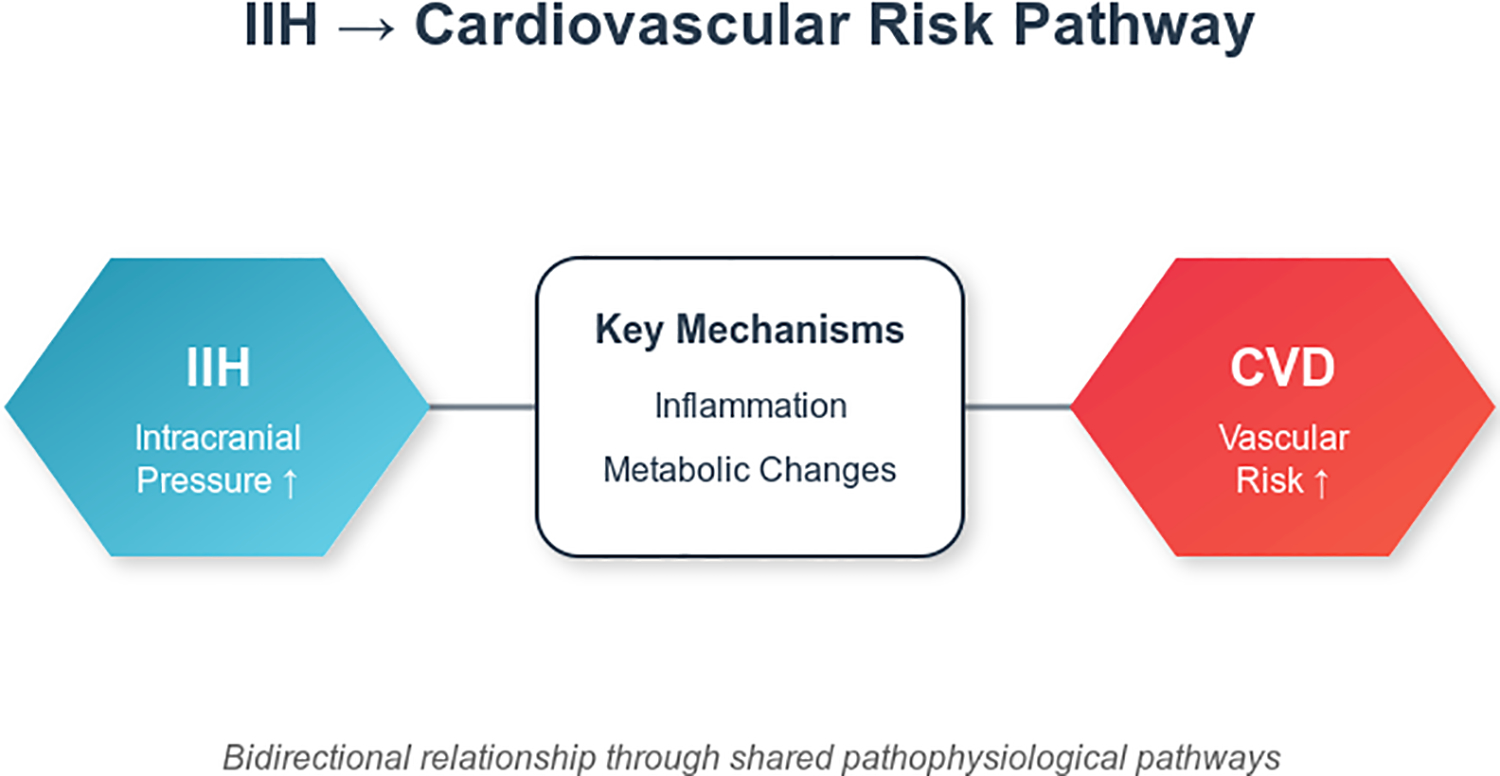
IIH and CVD Risk Pathway.

In our obesity-adjusted analysis, we have uncovered several significant findings that advance our understanding of how IIH influences CVD outcomes. Our primary analysis demonstrated that IIH independently raises CVD risk, as we observed consistent risk elevations (RR= 1.54 to 2.28) across CVD outcomes in our obesity-matched cohorts. Perhaps our most striking finding was the synergistic interaction between IIH and obesity, we found a 6.19-fold increased risk of composite CVD events (95% CI: 4.58–8.36, p<0.001) in obese IIH patients compared to non-obese controls. Through our modelling, we also discovered a metabolic relationship: non-obese IIH patients showed CVD risks comparable to obese controls which is significantly higher than non-obese controls (RR 2.18, 95% CI: 1.41–3.39, p<0.001). We were particularly intrigued by the paradoxical relationship we observed with type 2 diabetes risk which was elevated in obese IIH patients but reduced in non-obese IIH patients compared to obese controls, suggesting more complex metabolic mechanisms than previously recognized (Figure 5).

The consistent elevation of risk ratios across Models 1 and 4, which compare IIH patients to controls within the same obesity strata, strongly suggests a distinct pathophysiological process intrinsic to IIH that exacerbates cardiovascular vulnerability. This finding aligns with emerging research on the neuroendocrine and metabolic perturbations in IIH. Recent metabolomic profiling by O’Reilly MW et al [8]. revealed a unique signature of altered androgen metabolism in CSF of IIH patients, characterized by elevated levels of testosterone and androstenedione. This androgen excess may represent a crucial link between IIH and cardiovascular risk through multiple mechanisms, including vascular dysfunction, inflammatory modulation, and metabolic dysregulation. Duckles and Miller [61] demonstrated that testosterone could induce vasoconstriction through both genomic and non-genomic pathways, potentially contributing to hypertension and altered cerebrovascular autoregulation in IIH.

The chronic elevation of in ICP is a characteristic of IIH may have direct and indirect effects on cardiovascular functions. Recent work by Wardlaw et al. [62] on the glymphatic system and intracranial fluid dynamics suggests that altered CSF flow and clearance in IIH may impair the removal of metabolic waste products from the brain. This accumulation of potentially toxic metabolites could exacerbate oxidative stress and vascular inflammation, contributing to the observed CVD risk.

The striking risk elevations observed in Model 2 (Obese IIH vs Non-obese Control) reveal a synergistic interaction between IIH and obesity that amplifies CVD risk beyond the sum of their individual effects. This synergy likely arises from the convergence of multiple pathophysiological processes, including adipokine dysregulation, neuroendocrine activation, and hemodynamic alterations. Recent work by Hornby et al. [63] demonstrates that IIH patients exhibit a distinct adipokine signature, with particularly elevated CSF leptin levels. The combination of systemic and central adipokine dysregulation may create a uniquely pro-inflammatory and pro-thrombotic state. Moreover, the evidence by Markey K et al. [64] suggests that IIH patients may have altered cortisol metabolism, potentially exacerbating the metabolic and CVD consequences of obesity-related hypothalamic-pituitary-adrenal axis dysfunction.

The paradoxical findings regarding type 2 diabetes risk in our study—elevated in obese IIH patients but reduced in non-obese IIH patients compared to obese controls—challenge our current understanding of metabolic risk in IIH. This observation may be explained by the concept of “metabolic flexibility” proposed by Goodpaster and Sparks [65]. In non-obese IIH patients, the altered androgen metabolism and potential changes in adipose tissue function may confer a degree of metabolic protection. The evidence by Mariniello et al. [66] on androgen effects on adipose tissue suggests that certain androgen profiles can enhance insulin sensitivity and improve glucose uptake in adipocytes. The specific androgen milieu in IIH may thus have differential effects depending on the overall metabolic context. Conversely, in obese IIH patients, this potential metabolic benefit may be overwhelmed by the profound insulin resistance and chronic inflammation associated with obesity. The interaction between obesity-related metabolic dysfunction and IIH-specific neuroendocrine perturbations may create a “perfect storm” for accelerated progression to type 2 diabetes [66].

Our findings necessitate a paradigm shift in the approach to cardiovascular risk management in IIH patients. We propose a multi-tiered strategy that includes enhanced risk stratification, targeted interventions, personalized metabolic management, and neuroendocrine modulation. The development of IIH-specific CVD risk calculators that incorporate novel biomarkers such as CSF androgen levels, adipokine profiles, and measures of intracranial pressure dynamics could significantly improve risk assessment in this population. Exploration of IIH-specific pharmacological interventions that address the unique pathophysiology of CVD risk in this population is warranted. For example, the potential use of selective androgen receptor modulators (SARMs) to mitigate the adverse cardiovascular effects of androgen excess while preserving potential metabolic benefits merits investigation.

Future research directions should include longitudinal studies employing advanced imaging techniques to elucidate the temporal relationship between IIH onset, progression, and cardiovascular remodelling. Multi-omics approaches integrating genomics, transcriptomics, and metabolomics could unravel the molecular mechanisms underlying the observed synergy between IIH and obesity in cardiovascular risk.

Interventional trials exploring the cardiovascular impact of IIH-specific treatments, including the potential cardioprotective effects of CSF diversion procedures or novel pharmacological agents targeting ICP regulation, are crucial. Additionally, investigation of sex-specific aspects of cardiovascular risk in IIH is essential, given the strong female predominance of the condition and the potential interaction with sex hormones.

The findings from our study reveal a complex, multifaceted relationship between IIH, obesity, and CVD risk that challenges existing paradigms and opens new frontiers in personalized medicine. The independent risk conferred by IIH, the synergistic effects with obesity, and the paradoxical metabolic findings underscore the need for a nuanced, mechanism-based approach to cardiovascular risk management in this unique patient population. As we continue to unravel the intricate pathophysiology of IIH, we move closer to developing targeted interventions that may not only alleviate the neurological symptoms of the condition but also mitigate its long-term cardiovascular consequences. The implications of our findings extend beyond IIH, offering potential insights into the broader interplay between neuroendocrine function, metabolic regulation, and cardiovascular health. The methodology of our paper has several limitations, at first the approach assumes that the HR and the values provided from the original data and the HR for obesity remains constant over the 13-year period and its applicable to both the IIH group and control group.

Secondly, it assumes that the effect of obesity on the events is independent of IIH status in each patient. Thirdly, the predicted events are based on the average HR for obesity from the current literature, which may not be fully representative of the study population in larger populations or another cohort. Also, the adjusted for IIH independent from obesity should be interpreted with caution, as it is an estimation based on the available data and assumptions. To further validate the findings, it would be better to perform tailored individual-level data analysis based on BMI subgroup analysis and sensitivity tests for IIH patients and counting for other potential cofounding variables in the cohort. Additionally, conducting a prospective study that directly compares IIH patients with normal weight controls would provide more comprehensive evidence for the independent effect of IIH on the proposed events.

## 5. Conclusions

Through our findings, we have established compelling evidence that IIH independently contributes to CVD risk beyond obesity alone. Our statistical modelling has revealed that IIH operates through both independent and obesity-synergistic pathways to elevate CVD risk. We consistently observed elevated risks across our obesity-stratified models, leading us to believe that IIH involves an intrinsic pathophysiological process that worsens CVD outcomes vulnerability. These findings align with emerging research on neuroendocrine dysregulation in IIH. Based on our results, we strongly advocate for a fundamental shift in IIH management to include comprehensive CVD risk assessment and mitigation. We believe developing IIH-specific CVD risk assessment tools and targeted interventions should be a priority. While we acknowledge the limitations of our study, including our assumptions about hazard ratio consistency and obesity effects, we have established a crucial foundation for future studies. We recommend prospective studies comparing IIH patients with normal-weight controls and deeper investigation of underlying mechanisms through multi-omics approaches. Our findings have significant implications for both clinical practice and future research in IIH management.

## Figures and Tables

**Table 1:** Baseline Characteristics of the Included Individuals in the Original Study.

Variable	Number, (%)	
	Women With IIH (Exposed Group)	Women Without IIH (Control Group)
**Population**	2760 (9.2)	27 125 (90.8)
**Incident Cohort**	1331 (48.2)	12 679 (46.7)
**Population Aged < 60 y**	2709 (98.1)	25 811 (95.2)
**Age, Median (IQR), y**	32.1 (25.62–42.00)	32.1 (25.71–42.06)
**Townsend Deprivation Quintile**		
1 (Least deprived)	361 (13.1)	4268 (15.7)
2	381 (13.8)	4397 (16.2)
3	532 (19.3)	5174 (19.1)
4	538 (19.5)	5122 (18.9)
5 (Most deprived)	454 (16.5)	4134 (15.2)
Missing data	494 (17.9)	4030 (14.9)
**Smoking Status**		
Nonsmoker	1284 (46.5)	15 058 (55.5)
Ex-smoker	502 (18.2)	4573 (16.9)
Smoker	849 (30.8)	6134 (22.6)
Missing data	125 (4.5)	1360 (5.0)
BMI, median (IQR)	34.80 (29.30–40.30)	34.30 (29.00–39.70)
**Body Mass Index (BMI)**		
<25	246 (8.9)	2561 (9.4)
25–30	416 (15.1)	4203 (15.5)
>30	1728 (62.6)	16 514 (60.9)
Missing data	370 (13.4)	3847 (14.2)
Current lipid prescription	180 (6.5)	1572 (5.8)
Migraine	580 (21.0)	3247 (11.9)
**Outcomes at Baseline**		
**Heart Failure**	8 (0.3)	58 (0.2)
**IHD**	35 (1.3)	245 (0.9)
**Ischemic Stroke / TIA**	46 (1.7)	189 (0.7)
**Hypertension**	380 (13.8)	2500 (9.2)
**Type 2 Diabetes Mellitus**	130 (4.7)	1425 (5.2)

Abbreviations: IIH= Idiopathic Intracranial Hypertension; IQR= Interquartile Range; BMI= Body Mass Index; IHD= Ischemic Heart Disease; TIA= Transient Ischemic Attack.

**Table 2:** Risk Contribution Calculations According to Different Hazard Regression Models.

Outcome	Women With IIH (Exposed Group)	Women Without IIH (Control Group)	P-value
**Composite CVD**			
Population, No.	2613	26 356	NA
Outcome events, No. (%)	68 (2.5)	328 (1.2)	NA
Person-years	12 809	132 930	NA
Crude incidence rate per 1000 person-years	5.31	2.47	NA
Follow-up, median (IQR), y	3.50 (1.34–7.11)	3.72 (1.51–7.39)	NA
Adjusted HR (95% CI)			
Model 1	**2.15 [1.66 – 2.79]**	NA	**<.001** **
Model 2	**6.19 [4.58 – 8.36]**	NA	**<.001** **
Model 3	0.76 [0.50 – 1.15]	NA	0.2
Model 4	**2.18 [1.41 – 3.39]**	NA	**<.001** **
***Heart Failure***			
Population, No.	2735	26 989	NA
Outcome events, No. (%)	17 (0.6)	78 (0.3)	NA
Person-years	13 445	136 357	NA
Crude incidence rate per 1000 person-years	1.26	0.57	NA
Follow-up, median (IQR), y	3.58 (1.38–7.26)	3.77 (1.52–7.50)	NA
Adjusted HR (95% CI)			
Model 1	**2.21 [1.31 – 3.74]**	NA	**<.001** **
Model 2	**5.75 [3.17 – 10.42]**	NA	**<.001** **
Model 3	0.91 [0.42 – 1.97]	NA	0.81
Model 4	**2.37 [1.04 – 5.39]**	NA	**0.04** *
***IHD***			
Population, No.	2698	26 749	NA
Outcome events, No. (%)	27 (0.9)	131 (0.5)	NA
Person-years	13 216	134 521	NA
Crude incidence rate per 1000 person-years	2.04	0.97	NA
Follow-up, median (IQR), y	3.56 (1.37–7.20)	3.73 (1.51–7.42)	NA
Adjusted HR (95% CI)			
Model 1	**2.10 [1.39 – 3.17]**	NA	**<.001** **
Model 2	**3.76 [2.42 – 5.85]**	NA	**<.001** **
Model 3	1.17 [0.68 – 1.99]	NA	0.57
Model 4	**2.09 [1.20 – 3.65]**	NA	**<.01** *
***Stroke/TIA***			
Population, No.	2674	26 755	NA
Outcome events, No. (%)	40 (1.5)	181 (0.7)	NA
Person-years	13 115	135 271	NA
Crude incidence rate per 1000 person-years	3.05	1.34	NA
Follow-up, median (IQR), y	3.51 (1.34–7.17)	3.76 (1.52–7.47)	NA
Adjusted HR (95% CI)			
Model 1	**2.28 [1.62 – 3.21]**	NA	**<.001** **
Model 2	**3.93 [2.73 – 5.66]**	NA	**<.001** **
Model 3	1.37 [0.89 – 2.09]	NA	0.15
Model 4	**2.36 [1.51 – 3.67]**	NA	**<.001** **
***Hypertension***			
Population, No.	2232	23 566	NA
Outcome events, No. (%)	148 (6.2)	1059 (4.3)	NA
Person-years	10 505	115 800	NA
Crude incidence rate per 1000 person-years	14.09	9.15	NA
Follow-up, median (IQR), y	3.20 (1.26–6.40)	3.48 (1.43–6.94)	NA
Adjusted HR (95% CI)			
Model 1	**1.54 [1.30 – 1.83]**	NA	**<.001** **
Model 2	**3.22 [2.68 – 3.86]**	NA	**<.001** **
Model 3	**0.77 [0.61 – 0.97]**	NA	**0.03** *
Model 4	**1.61 [1.26 – 2.05]**	NA	**<.001** **
***Type 2 Diabetes***			
Population, No.	2510	24 901	NA
Outcome events, No. (%)	120 (4.6)	799 (3.1)	NA
Person-years	12 300	125 947	NA
Crude incidence rate per 1000 person-years	9.76	6.34	NA
Follow-up, median (IQR), y	3.49 (1.34–6.94)	3.62 (1.47–7.27)	NA
***Adjusted HR (95% CI)***			
Model 1	**1.54 [1.27 – 1.86]**	NA	**<.001** **
Model 2	**6.14 [4.90 – 7.70]**	NA	**<.001** **
Model 3	**0.40 [0.28 – 0.57]**	NA	**<.001** **
Model 4	**1.59 [1.09 – 2.32]**	NA	**0.02** *

* Denotes statistical significance,

** Denotes high statistical significance

Abbreviations: IIH= Idiopathic Intracranial Hypertension; CVD= Cardiovascular Disease; IQR= Interquartile Range; IHD= Ischemic Heart Disease; CI= Confidence Interval.
